# News from Australia

**Published:** 1918-07-13

**Authors:** 


### NEWS FROM AUSTRALIA.

(FROM OUR AUSTRALIAN CORRESPONDENT.)

### The Doctors' "Strike" and State Action.

OUR Australian correspondent writes: The doctors' "strike" is still proceeding, although public opinion is on the side of the Friendly Societies. The Societies, except those that have already formed dispensaries or medical institutes, are feeling the strain on their funds. The Bowser (State) Ministry, through the Premier, three times asked the B.M.A. to submit its claims to friendly arbitration, and was as often refused. Then a Bill to compel arbitration was sought to be drafted. This took time and consideration, as it became more evident that compulsion of the doctors was impossible. A sudden change of Ministry taking place in mid-March added to the difficulties, but the Lawson Ministry adopted the Bowser Bill, and on March 27 it was passed with brief discussion. No compulsory provisions have been introduced, for the object of the Bill was to bring the parties together in a spirit of conciliation. The new Bill provides for a Board of eleven—five delegates from each side. The Labour member was the author of the amendment that no barrister or solicitor should be allowed to appear before the Board. This was carried. Unless repealed, the Act is now law for the term of the war and six months afterwards.

The point most resented by the people is that the B.M.A. should have broken its pledge to observe a truce which it distinctly asked for—not to reopen the question of increased fees from lodges during the term of the war. To this resentment is due the growth of the dispensary or medical institute system, which is rapidly extending not only in and around the metropolitan area, but throughout the country. It is quite likely that after the war the fee of 20s. per annum per member asked for by the doctors will be paid in the towns, but improbable that the 26s. demanded for country members will ever be forthcoming.

Should the renewed efforts towards conciliation of the two parties be fruitless, it is thought by many that Nationalisation of Medicine would be the only possible next step. This idea has long been very favourably regarded in Australia by many leading medical practitioners.

On April 23 the State Cabinet, after reading a report from the Premier (Mr. Lawson) upon the unlikelihood of the B.M.A. altering its decision not to send delegates to the proposed conference between that body and the Friendly Societies, decided to appoint a Royal Commission to investigate the matter. In announcing the decision, the Premier said that he had expressed amazement at the action of the B.M.A., in defying an Act of Parliament, refusing to submit to constitutional authority, and even refusing to negotiate on the merits of the dispute. Judge Wasley has undertaken the inquiry, which will probably take him to Sydney, where the Friendly Societies, a few years ago, conceded what the doctors in Victoria are now asking for. The Government has stipulated that the matter be conducted with the utmost dispatch. The question of counsel appearing—which is warmly objected to by the F.S. Association—was left to the Commission's decision. The president is also asked to determine the terms and conditions of agreements, including doctors' remuneration and the method of arriving at such payment both for ordinary and special conditions.

The Commission's first sitting was held on May 8, and on May 20 Mr. Owen Dixon explained that the B.M.A. retained its attitude as to the unfitness of settling such a dispute by arbitration.

### Medical Institutes.

These continue to increase. Ballarat has decided to appoint three medical officers at a salary of £1,000 per annum, with accouchements and operations extra. The salary was first fixed at £800, but was increased in view of the present high cost of living.

Maryborough, a small town, has just appointed a medical officer at £800 per annum, with the extras. They had five applications, and selected a young man from New South Wales but a graduate of the Melbourne University.

